# Enhancing particle swarm optimization based on optical computing mechanism: application to dyslexia detection

**DOI:** 10.3389/frai.2025.1731997

**Published:** 2026-01-30

**Authors:** Nermine Mahmoud, Mohamed Abd Elaziz, Abdelghani Dahou, Mohammad Ghatasheh, Ibrahim A. Fares, Mohammed Azmi Al-Betar, Ahmed A. Ewees

**Affiliations:** 1 Faculty of Social and Human Sciences, Galala University, Suez, Egypt; 2 Artificial Intelligence Research Center (AIRC), College of Engineering and Information Technology, Ajman University, Ajman, United Arab Emirates; 3 School of Computer Science and Technology, Zhejiang Normal University, Jinhua, China; 4 Department of Computer Science, Middle East University, Amman, Jordan; 5 Department of Cybersecurity, College of Engineering and Information Technology, Buraydah Private Colleges, Buraydah, Saudi Arabia; 6 Department of Mathematics, Faculty of Science, Zagazig University, Zagazig, Egypt; 7 Center of Excellence in Precision Medicine and Digital Health, Department of Physiology, Geriatric Dentistry and Special Patients Care Program, Faculty of Dentistry, Chulalongkorn University, Bangkok, Thailand; 8 Department of Computer, Damietta University, Damietta, Egypt

**Keywords:** dyslexia, global optimization, metaheuristic, optical computer, particle swarm optimization (PSO)

## Abstract

**Introduction:**

This study presents a modified version of Particle Swarm Optimization (PSO) using an all-optical computational update mechanism. The primary innovation and objective of this collaboration aimed to leverage the inherent properties of coherent optical systems, including specialized complex-domain computation and nonlinear light-matter interactions, to enhance the exploration and exploitation of the search space process for particles.

**Methods:**

To assess the performance of the developed model, it was compared with traditional PSO to solve the CEC benchmark functions. Furthermore, it was applied to enhance the detection of dyslexia using the eye-tracking dataset (ETDD).

**Results:**

The comparison between OPSO and other techniques established its high ability to enhance the detection of dyslexia over traditional techniques.

**Discussion:**

The use of an all-optical computational update mechanism demonstrated enhanced performance in both benchmark optimization problems and dyslexia detection tasks.

## 1 Introduction

Artificial intelligence (AI), particularly modern deep learning (DL), has transformed an extraordinary range of application domains over the past decade. Breakthroughs in computer vision, natural-language processing (NLP), speech recognition, and reinforcement learning have moved the performance of automated systems beyond human-level on benchmark tasks such as ImageNet image classification (LeCun et al., 2015), conversational language modeling (Brown et al., 2020), and board-game playing (Silver et al., 2018). In health and life sciences, deep neural networks already match or surpass specialist clinicians in skin cancer recognition (Esteva et al., 2017), chest-X-ray triage (Rajpurkar et al., 2017), and diabetic retinopathy screening (Gulshan et al., 2016). These successes illustrate the general ability of AI methods to learn complex, high-dimensional patterns from raw or minimally processed data, providing objective, reproducible, and scalable decision support in settings where conventional analyses remain labor-intensive or inconsistent (Topol, 2019).

A rapidly growing subfield applies AI to neuro-cognitive assessment. Automated analysis of speech, handwriting, and physiological signals has begun to aid the early detection of neurodevelopmental and neurodegenerative disorders such as autism spectrum disorder, hyperactivity disorder, Parkinson's disease, and Alzheimer's disease (Probol and Mieskes, 2024; Nafisah et al., 2025; Gallo-Aristizabal et al., 2025). Within this field, reading disabilities, and dyslexia in particular, represent a compelling target for AI-based diagnostics.

Dyslexia is a specific learning disability of neurobiological origin that impairs reading and language processing skills. It affects an estimated 5%–10% of the population worldwide ( 7% of children, accounting for up to 80% of learning disorders). It is characterized by persistent difficulties in word recognition, decoding, and reading fluency despite adequate intelligence and education (Tiwari et al., 2025; Toki, 2024). Early identification is critical: timely evidence-based intervention can exploit neural plasticity to improve literacy outcomes and mitigate long-term academic and socio-emotional consequences (El Hmimdi et al., 2024). However, conventional diagnostic procedures rely on standardized reading batteries and clinician observation—methods that are time-consuming, subjective, and often unavailable in many educational settings, leading to delayed or inaccurate diagnoses (Snowling, 2013).

Eye-tracking provides a non-invasive, real-time record of visual attention during reading, capturing spatiotemporal gaze trajectories at millisecond resolution. Dyslexic readers consistently produce more frequent and longer fixations, shorter saccades, and an increased number of regressions relative to typically developing peers (Yin et al., 2025). Because these metrics directly reflect underlying cognitive processing demands, eye-movement data furnish a rich, quantitative substrate for automated dyslexia detection.

Machine-learning (ML) approaches leveraging handcrafted eye-movement features have achieved promising performance. Jothi Prabha and Bhargavi (2022) and El Hmimdi et al. (2024) extracted 168 temporal, spatial, and frequency descriptors, applied recursive feature elimination (RFE), and trained a support-vector machine (SVM) that reached 95.6% accuracy. Comparable results have been reported with principal-component analysis and SVM (Nerušil et al., 2021), evolutionary optimization plus SVM (Jothi Prabha and Bhargavi, 2022), *k*-nearest neighbors and random forests, provided that irrelevant or redundant variables are pruned (Prabha and Bhargavi, 2020). Nonetheless, performance varies with dataset size and reading paradigm, occasionally dropping to ~80% accuracy on smaller or more heterogeneous samples, underscoring challenges of generalization.

DL methods aim to circumvent manual feature engineering by learning hierarchical representations directly from raw gaze sequences. Nerušil et al. (2021) proposed a convolutional neural network (CNN) that ingests (*x, y*) time-series and implicitly captures fixation–saccade dynamics. The recently released ETDD70 dataset—70 Czech children, 35 with dyslexia—enabled (Sedmidubsky et al., 2024) to obtain ~90% accuracy with a CNN, while Gomolka et al. (2024) achieved 97.7% using a long short-term memory (LSTM) network. Transformer architectures with multi-head attention have pushed reported accuracies even higher (~99%) in tightly controlled experiments (Priyasri and Devi, 2025). Conversely, when trained on a large and diverse corpus of >4, 000 reading traces, a CNN delivered more modest but still encouraging precision and recall of 77%–80% (El Hmimdi et al., 2024), highlighting persistent risks of overfitting and data-shift.

With the advantages achieved by the previous studies, they still have limitations. This motivated us to develop an alternative AI technique that improves the performance of particle swarm optimization (PSO) (Kennedy and Eberhart, 1995). This modification was conducted using the optical computing mechanism (OCM) (Zuo et al., 2019; Lu and Saleh, 1990). In general, OCM depends on photonics that improve and accelerate the computation of complex matrix-vector multiplication. Therefore, the OCM has advantages over electronics, such as high computation speed and parallelism (Kitayama et al., 2019). The increasing complexity of data manipulation techniques and the size of data lead to an increase in demand for highly integrated, scalable optical hardware that is ultracompact and consumes less energy. Therefore, the advantages of ultracompact size lead to the establishment of large, compact computing units. This is considered the main component in optical-artificial-intelligence computers (Feldmann et al., 2021; Kues et al., 2017). This inspired us to simulate the operation of an optical computing mechanism and integrate it with a metaheuristic (MH) technique named PSO. The primary reason for using PSO rather than other MH methods is its broad applicability across diverse applications and its role as the basis for many other MH techniques.

The developed model, named optical PSO (OPSO), begins by generating a set of solutions. Then, it computes the fitness value and determines the best personal and global best values. Next, we simulated the processing of particle-state information through an optical device by encoding the input and performing complex mixing and nonlinearity. The encoding of the input is formulated by computing the optical of the input vector (i.e., current particle, best personal, and global best particle) based on the magnitude and 2π-scaled. The input signal is applied to a coherent linear system using the complex weight matrix, thereby emulating the physical diffraction and interference. Next, a nonlinear saturation of light in a nonlinear crystal is simulated. Thereafter, the optical output is referred to as the optical delta, which is computed as the real part of the nonlinear saturation. Then, the adaptively mixed delta is computed, which depends on the combination of the optical and classical PSO terms using a decaying mixing ratio. Finally, the particle's velocity is updated using the adaptively mixed delta, and a new particle is generated based on the updated velocity. First, the stop conditions are checked to determine whether they are met; then the best solution is used as the output. To assess the performance of the developed model, we applied it to the CEC2019 benchmark functions and used it as a feature selection method to enhance the detection of Dyslexia.

The main contributions of this study can be summarized as follows:

- Implement the optical computing of photons and use it to enhance the performance of PSO.
- Develop an alternative dyslexia detection technique based on the proposed OPSO.
- Assess the applicability of proposed OPSO by using CEC2019 benchmark and different tasks of dyslexia data.

The rest of this study is organized as follows: In Section 2, related work of PSO variants is introduced. In Section 3, the background of traditional Particle Swarm Optimization (PSO) is presented. Section 4 presents the steps of the developed Optical OPSO. Section 5 introduces the experimental results of the proposed model to handle global optimization problems. Whereas Section 6 shows the results of the proposed OPSO to detect dyslexia. The conclusion and future works are given in Section 7.

## 2 Literature review of PSO variants

A recent study on PSO introduced a wide range of variants that adjust the learning strategy, swarm topology, and hybridization mechanisms to improve the balance between exploration and exploitation, increase robustness, and better adapt to specific problem types (Chauhan et al., 2025). Several PSO variants have been developed to automatically adjust the inertia weight, learning factors, or velocity limits to control the search process (Shami et al., 2022), providing parameter control and the bare bones of PSO. ECLPSO and ACLPSO, for example, use adaptive learning probabilities, perturbation-based exploitation, and dimension-wise parameter adaptation to enhance both exploration and convergence compared with the original CLPSO (Lin et al., 2019; Yu and Qiao, 2021). A bare-bones PSO (BBPSO) remains an important minimalist variant that removes velocities and samples positions from probability distributions. It has been extended with deep memory and scale-matrix adaptation to achieve higher precision while maintaining diversity (Song et al., 2021; Sun et al., 2023).

For Learning-Strategy PSO (CLPSO Family; Lin et al., 2019; Liang et al., 2006), one powerful variation supports each particle to learn from the personal-best positions of several peers rather than depending on a single global or local best. Comprehensive learning PSO (CLPSO) enables every particle to learn. This broader information sharing supports diversity and lowers early convergence. Following this concept, ECLPSO, ACLPSO, and CLPSO-OPR introduce adaptive learning probabilities, perturbation operators, and recombination rules in subsequent iterations to more effectively balance broad-area exploration with focused local search (Liang et al., 2006). Recent polls emphasize these learning-oriented PSOs as a significant current trend because they alter how information is shared within the swarm rather than merely tweaking parameter settings (Liang et al., 2006; Chen et al., 2024).

In multiswarm and cooperative PSO, dynamic multi-swarm PSO (DMS-PSO) divides the population into several small sub-swarms that are often regrouped, giving each sub-swarm local autonomy while allowing periodic information exchange to avoid local optima (Zhao et al., 2008). Later research combined DMS-PSO with local search or harmony search to enhance local accuracy and large-scale performance, especially on multimodal benchmarks (Zhao et al., 2011). Many surveys highlight these multi-swarm and collaborative systems as successful in difficult terrain, as they preserve diversity without completely sacrificing convergence speed.

Increasingly, hybrid PSO studies combine BBPSO or classic PSO with problem-specific techniques, including local search, adaptive mutation, and mutual-information-based assessment (Grazioso et al., 2025). For example, a basic PSO with mutual-information-guided feature selection introduces adaptive flip mutation and leader-update rules, enabling efficient dimensionality reduction and competitive classification performance on benchmark datasets. Deep memory BBPSO (DMBBPSO) introduces a unique memory topology and pruning method that allows particles to leverage extensive historical information while eliminating obsolete data, thereby supporting both extensive exploration and high-precision local search (Grazioso et al., 2025). These PSO variants are summarized in Table 1.

**Table 1:** Variants of the PSO algorithm.

**Variant**	**Main update**	**Advantages**	**Limitations**
BBPSO (2003) (Sun et al., 2023)	Removes velocity; updates positions via Gaussian sampling.	Simple structure; improved accuracy and efficiency compared with standard PSO.	Prone to premature convergence; sensitive to sampling distribution.
SMA-BBPSO (2010s) (Sun et al., 2023)	Introduces scale-matrix adaptation and heavy-tailed sampling (e.g., *t*-distribution).	Greater diversity; better escape from local optima.	More parameters; scale-matrix tuning depends on the problem.
PBBPSO (2010s) (Sun et al., 2023)	Adds pair-wise interaction rules for cooperative position updates.	Supports swarm diversity; slows overly fast convergence.	Higher communication cost; limited benefit on simpler problems.
DMBBPSO (2023) (Sun et al., 2023)	Uses deep memory topology and pruning of outdated historical data.	High-precision local search with strong global exploration.	Complex memory management; risk of retaining misleading history.
CLPSO (2006) (Liang et al., 2006)	Dimension-wise learning from multiple particles' personal bests.	High diversity; strong results on multimodal functions.	Higher computational load; slower on unimodal tasks.
ECLPSO (pre-2021) (Yu and Qiao, 2021)	Adds perturbation-based exploitation and adaptive learning probabilities.	Better exploitation and improved convergence over CLPSO.	Extra parameters; still underperforms on some complex landscapes.
ACLPSO (2021) (Yu and Qiao, 2021)	Adaptive velocity limits, inertia weights, learning factors, and probabilities.	Enhanced global search ability; robust convergence on benchmarks.	Increased complexity; performance sensitive to adaptation rules.
CLPSO-OPR (2020s) (Chen et al., 2024)	Integrates optimal particle recombination into CLPSO.	Enhanced exploration reduces premature convergence.	The recombination step may increase computation and need tuning.
DMS-PSO (2008, 2011) (Zhao et al., 2008, 2011)	Uses many small sub-swarms with dynamic regrouping; hybrid-friendly.	Effective for multimodal and large-scale problems.	More parameters increase computational cost.
MI-BBPSO (2021) (Song et al., 2021)	Uses mutual-information evaluation with adaptive flip mutation.	Produces compact feature sets; competitive classification accuracy.	Tailored to feature selection; it depends on the classifier and MI estimates.

## 3 Background of particle swarm optimization

The particle swarm optimization (PSO) algorithm is considered one of the most popular and efficient metaheuristic techniques. In general, PSO simulates the behavior of particles in nature during the food search. This includes the communication between populations and how they explore the search space.

To achieve the process of finding the food, the position and velocity of each particle are updated. The first step in PSO is to generate the initial value for the velocity and position of a set of *N* particles. Then, the fitness value for each particle is computed, and the best personal particle (*X*_*pi*_) and the global best particle (*X*_*g*_) are determined. The next process is to update the velocity of each particle (*v*_*i*_) using Equation 1.


$$\begin{array}{l} v_{i}\left(t+1\right)=w\times v_{i}\left(t\right)+c_{1}rand\left(Xpi-X_{i}\left(t\right)\right)+c_{2}rand\left(X_{g}-X_{i}\left(t\right)\right) \end{array}$$ (1)

Where *c*_1_ and *c*_2_ refer to the weight coefficient of the best local and global positions, respectively. *w* is the inertia coefficient, which controls the influence of the previous velocity on the updated velocity. The next step after updating the velocity is updating the particle's position. *X*_*i*_ as in Equation 2.


$$\begin{array}{l} X_{i}\left(t+1\right)=X_{i}\left(t\right)+v_{i}\left(t+1\right) \end{array}$$ (2)

The process of updating the position is repeated until the stop conditions are reached. Algorithm 1 shows the steps of the PSO algorithm.

Algorithm 1 **Meta-training and meta-testing of TCPL.**

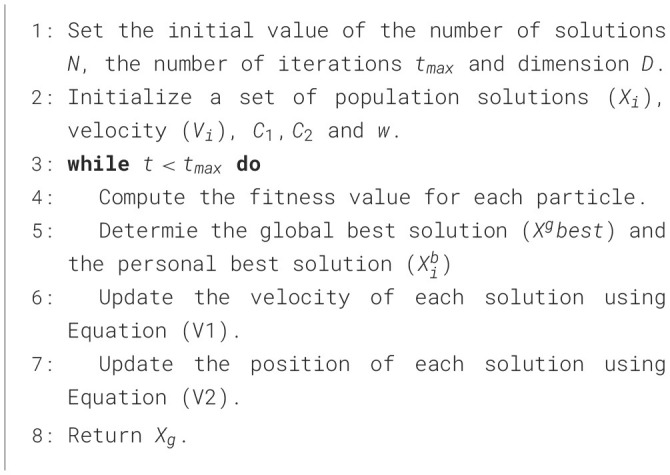

## 4 Optical particle swarm optimization

The proposed optical Particle Swarm Optimization (OPSO) algorithm is introduced in this section. The proposed OPSO embeds the mathematical properties of all-optical mechanisms within the standard PSO algorithm. This mechanism aims to leverage the inherent properties of coherent optical systems—specifically complex-domain computation and nonlinear light-matter interactions to enhance the search process for particles.

### 4.1 Mathematical formulation of the optical update mechanism

The main step is to update the optical delta (Δ_*opt*_), which represents the processing of particle-state information through a compact optical computing device. This is conducted through a set of steps, including complex input encoding, handling missing values and Nonlinearity, and a hybrid velocity update. The details of each step are provided in the following sections.

#### 4.1.1 Complex input encoding

The encoding process is conducted through three steps named normalizing, combining, and mapping the key state vectors of particle *i*—its current position (*x*_*i*_), personal best (*pbest*_*i*_), and global best (*gbest*)—into a complex-valued optical input vector (*Opt*_*in*_). This mapping depends on the magnitude of the real input vector for amplitude and a 2π-scaled, noise-injected version for the phase (ϕ):


$$\begin{array}{l} Opt_{in}=Amp\times e^{i\times \phi }=|In_{r}|\times e^{i\times 2\times \pi \left(In_{r}+r_{n}\right)} \end{array}$$ (3)

Furthermore, an adaptive Optical Noise (*opt*_*in*_) is linearly annealed over the iterations to balance initial exploration with later convergence. This results from maintaining the orthogonal storage of the search history, which influences the trajectory without directly altering the magnitude.

#### 4.1.2 Complex mixing and non-linearity

The next step is to transform the optical input using the process of a coherent linear system, followed by a non-linear medium:

For the linear optical mixing, the complex weight matrix (*W*_*opt*_), which is derived from a random unitary matrix, is applied to the input. This process is defined as


$$\begin{array}{l} z=W_{opt}\times Opt_{in} \end{array}$$ (4)

In general, Equation 4 represents the physical diffraction and interference that performs a matrix-vector multiplication. We used this process to support particles stuck at a local point.

In the non-linear saturation, the resulting vector is passed through a non-linear saturation function, formulating the physical saturation of light in a non-linear crystal:


$$\begin{array}{l} z_{nl}=\frac{z}{\left(1+\alpha \times |z{|}^{2}\right)} \end{array}$$ (5)

The real part of the saturated output is then scaled by an annealing factor to generate the final Δ_*opt*_. The non-linear saturation system acts as a soft-limiting regularizer. In the context of optimization, it suppresses extreme velocity updates while allowing smaller, precise adjustments to pass through linearly. This is crucial for fine-tuning the solution as the swarm approaches the global optimum.

### 4.2 Hybrid velocity update

The final update maintains stability by adaptively combining the optical result with the classical PSO steps. The Classical Delta (Δ_*C*_) is the standard cognitive and social contribution:


$$\begin{array}{l} \Delta_{C}=C_{1}\times r_{1}\times \left(P_{b}-X\right)+C_{2}\times r_{2}\times \left(P_{g}-X\right) \end{array}$$ (6)

The next process is to determine the movement direction through computing the combined delta (Δ_*Co*_) using an adaptive mixing ratio (*M*_β_), which shifts the emphasis from the optical update (exploration) to the classical update (exploitation). This process is defined in Equation 7.


$$\begin{array}{l} \Delta_{Co}=M_{\beta }\times \Delta_{opt}+\left(1-M_{\beta }\right)\times \Delta_{C} \end{array}$$ (7)

Thereafter, Δ_*Co*_ is used to update the velocity and position, incorporating a decreasing inertia weight (*w*_*inertia*_) and constriction factor (χ) to ensure convergence. This process is achieved using the following formula:


$$\begin{array}{l} V_{n}=\chi \times \left(w_{inertia}\times V_{i}+\Delta_{Co}\right), \end{array}$$ (8)

where *w*_*inertia*_ decreased from 0.7 to 0.3, and χ is updated as follows:


$$\begin{array}{l} \chi =\frac{2}{\left|2-\phi -\sqrt{\phi^{2}-4\phi }\right|},\phi =2.05 \end{array}$$ (9)

Where ϕ is a constant value that represents the stability parameter.

Finally, the position is updated using the new velocity value defined in Equation 10.


$$\begin{array}{l} X_{i}=X_{i}+V_{n} \end{array}$$ (10)

The algorithm also includes boundary absorption and an occasional reseeding mechanism (with *p* = 0.03) to maintain swarm diversity. The steps of the proposed OPSO are given in Algorithm 2.

Algorithm 2 **Meta-training and meta-testing of TCPL.**

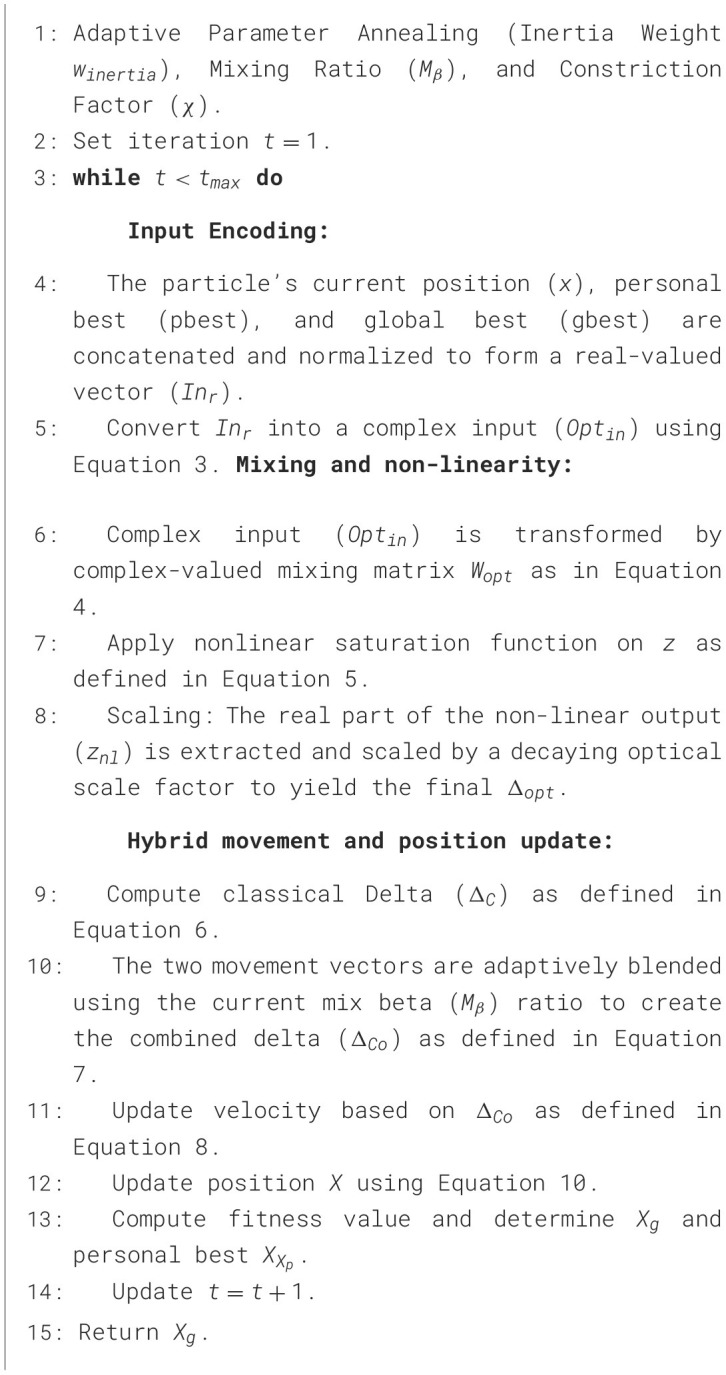

## 5 Experimental series 1: global optimization

This section presents an ablation study to evaluate the individual contributions of the components within the OPSO algorithm by comparing its performance against the standard PSO on the CEC2019 benchmark functions. The global experimental settings were configured to 1,000 iterations with a population size of 100. The comparative analysis reveals clear advantages of the proposed OPSO method in optimization accuracy as listed in Table 2.

**Table 2:** Results of the ablation study of OPSO.

	**Fitness**		**Std**		**Min**		**Max**		**Time**	
**Func**.	**OPSO**	**PSO**	**OPSO**	**PSO**	**OPSO**	**PSO**	**OPSO**	**PSO**	**OPSO**	**PSO**
F1	**4.51E+07**	8.66E+10	**3.62E+07**	8.86E+10	**3.52E+06**	1.53E+10	**1.45E+08**	4.29E+11	2.41E+01	**2.34E+01**
F2	**17.343**	6037.922	**0.000**	1591.923	**17.343**	1520.793	**17.343**	8535.809	1.619	**1.177**
F3	**12.702**	**12.702**	**0.000**	0.000	**12.702**	**12.702**	**12.702**	**12.702**	1.789	**1.331**
F4	**48.136**	223.305	**19.694**	232.919	17.912	**7.960**	**79.615**	945.137	1.388	**1.203**
F5	**1.255**	1.833	**0.119**	0.356	**1.098**	1.308	**1.586**	2.793	1.391	**1.189**
F6	7.792	**4.863**	**1.266**	2.057	4.636	**1.731**	**9.650**	9.938	8.678	**8.447**
F7	**96.028**	105.722	**60.651**	70.962	**0.254**	10.379	**211.521**	273.208	1.369	**1.178**
F8	**4.788**	4.914	0.674	**0.598**	**3.480**	3.703	6.010	**5.997**	1.368	**1.179**
F9	**2.390**	2.415	**0.019**	0.381	2.349	**2.339**	**2.420**	4.397	1.307	**1.127**
F10	**18.807**	19.344	4.900	**3.592**	**0.000**	**0.000**	20.344	**20.138**	1.393	**1.206**

Bold values indicate the best performance compared to the other values.

Across all benchmark functions, especially in F1 and F2, the OPSO algorithm performed, on average, better than classical PSO in terms of fitness. This suggests that, due to the properties of CEC2019 functions, OPSO more effectively explores the search space and converges more consistently toward diverse optima across different tests.

To examine the stability of the solutions, the standard deviation merit was used. OPSO across numerous functions yielded less variability in fitness results than PSO, indicating that the runs belonged to a consistent type of stability. This is an important factor to consider in practical applications, where the algorithm's performance must remain stable. This also indicated that the enhancements of the proposed method mitigate the random stochastic disturbances commonly encountered by traditional PSO.

The analysis of the minimum and maximum fitness values confirmed that OPSO kept the ranges between the best and worst results narrower for most functions. With PSO, there was greater variation, especially on problems where the function to be minimized was complex and the search space was high-dimensional. This supports the conclusion that OPSO strikes a better balance between exploration and exploitation and maintains the swarm's diversity, thereby reducing the occurrence of suboptimal solutions in difficult environments.

In terms of computational efficiency, OPSO exhibited performance similar to that of PSO. Although the optimization ability of OPSO is enhanced, this does not entail a significant additional computational burden. Thus, OPSO is suitable for large-scale problems and for applications where time is of the essence and efficiency and accuracy are equally important.

**Figure 1:**
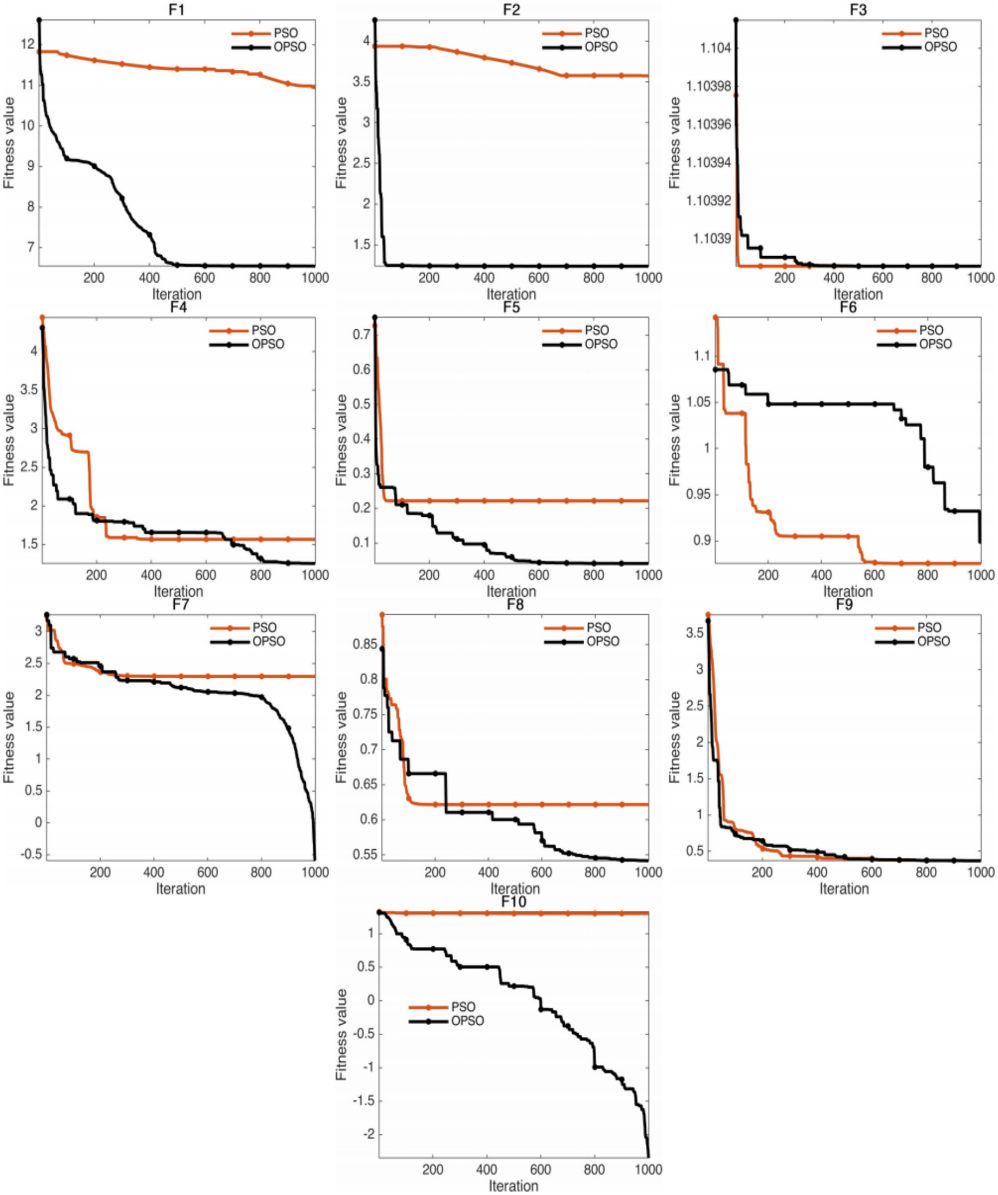
Convergence curves of OPSO and PSO for benchmark functions.

The convergence curves, as presented in Figure 1, indicate that OPSO reaches lower fitness values faster than PSO in most cases, especially on complicated test functions.

To summarize, these results demonstrate the effectiveness of OPSO as a warm-intelligence algorithm. The proposed method showed good accuracy, greater solution stability, and consistent convergence across the CEC2019 benchmarks.

## 6 Experimental series 2: dyslexia detection

### 6.1 Description of dyslexia dataset

To assess the performance of the proposed model, a public Eye-Tracking Dyslexia Dataset (ETDD70) (Sedmidubsky et al., 2024) is used. This dataset was collected from 70 Czech children (9–10 years, 50% dyslexic). Furthermore, the dataset contains three tasks of Czech reading named meaningful text (T1), pseudo-text (T4), and syllable reading (T5), administered under controlled conditions. Each task has two types of information: (1) images and (2) tabular data. Therefore, D1, D2, and D3 are images of T1, T4, and T5, respectively. Whereas D4, D5, and D6 are the tabular of T1, T4, and T5, respectively.

The noise-robust i2mc algorithm has been used to record and process eye movements (Hessels et al., 2017). The saccade amplitude and fixation duration are treated as tabular features collected from each participant and task. Meanwhile, the visual features (FixIma) are 2D images obtained using overlaying-colored ellipses that represent fixation location, size, and duration. In general, the tabular features for T1, T4, and T5 are 450, 34, and 34 features, respectively. These features are extracted from eye-tracking metrics, including fixation counts, saccade amplitudes, and durations. For the visual modality, FixIma images were generated for each task and resized to 224 × 224 pixels for input to the SwinV2 model.

### 6.2 Performance metrics

The performance of OPSO is computed using the following measures.

- Accuracy:

$$\begin{array}{c} \text{Accuracy}=\frac{TP+TN}{TP+TN+FP+FN} \end{array}$$ (11)

- Sensitivity:

$$\begin{array}{c} \text{Sensitivity}=\frac{TP}{TP+FN} \end{array}$$ (12)

Where *TP* and *TN* denote true positives and true negatives; *FP* and *FN* refer to false positives and false negatives, respectively.

Furthermore, the fitness value is used to evaluate the ability of the algorithm to balance between the classification error and the ratio of the selected features.

Moreover, the results of the proposed OPSO are compared with those of traditional PSO, Ant Colony Optimization (ACO) (Kashef and Nezamabadi-pour, 2015), Mountaineering team-based optimization (MTBO) (Faridmehr et al., 2023), Chaos Game Optimization (CGO) (Dahou et al., 2023), Differential Evolution (DE) (Wang et al., 2022), Success-History based Adaptive DE (LASHDE) (Tanabe and Fukunaga, 2014), and Arithmetic Optimization Algorithm (AOA) (Abualigah et al., 2021). The parameters of these algorithms are determined as in the original implementation. Following Sedmidubsky et al. (2024), we used the training and testing sets generated via 5-fold cross-validation, with folds divided by subject (i.e., 14 subjects in the testing fold). For the parameters of OPSO, such as Adaptive Parameter Annealing (Inertia Weight *w*_*inertia*_∈[0.7 − 0.3]), Mixing Ratio [*M*_β_ = 0.9 × (1−*t*/*t*_*max*_)], and reseeding mechanism (with *p* = 0.03).

### 6.3 Results and discussion

**Figure 2:**
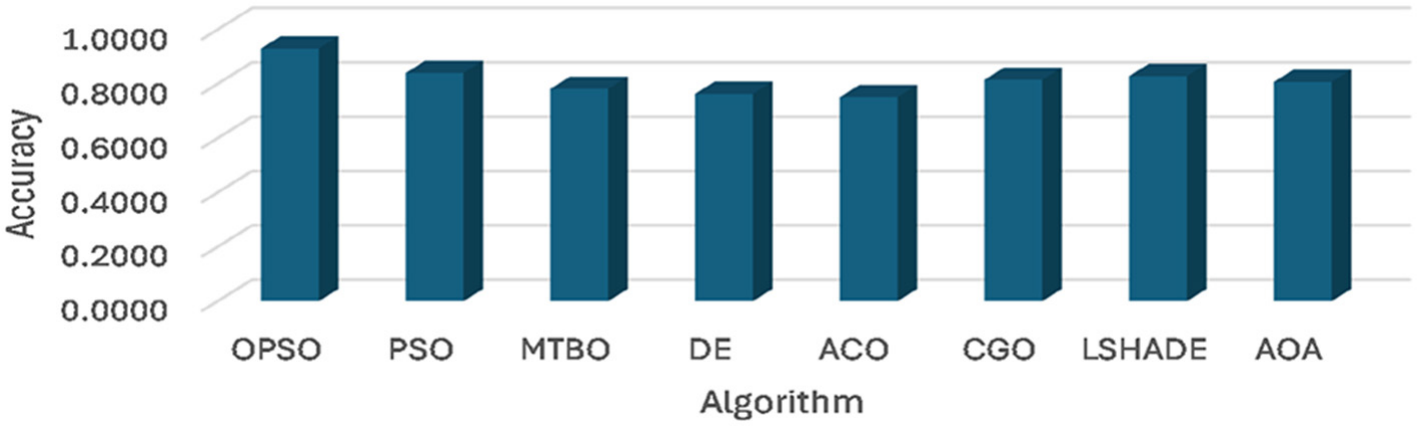
Average of accuracy obtained using OPSO and other algorithms.

**Figure 3:**
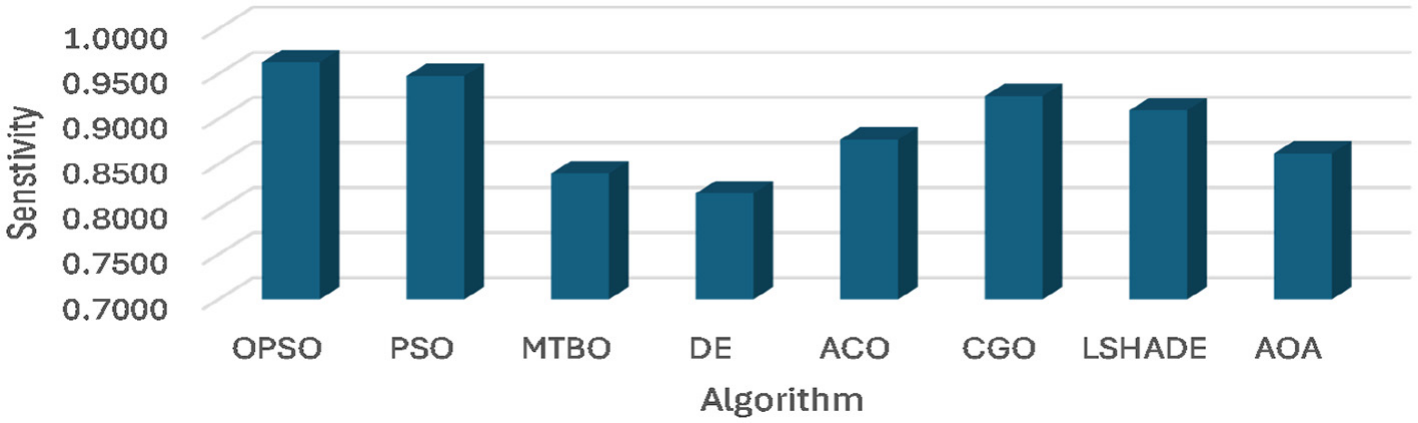
Average of sensitivity obtained using OPSO and other algorithms.

A comparison of the developed OPSO algorithm and others is given in Tables 3–5 and Figure 2. In general, Table 3 and Figures 2, 3 show the average accuracy and sensitivity of the algorithms among the datasets. From this table, it is evident that the OPSO algorithm demonstrated a significant superiority in mean accuracy, achieving the highest value in four of the six datasets (D1, D2, D3, and D4). The highest accuracy was achieved by OPSO in D2, with a value of 0.9591. However, the LSHADE algorithm achieved the highest accuracy in D5 (0.9286), while PSO and OPSO were equal in D6 (0.9143). Notably, the DE, ACO, MTBO, and CGO algorithms tended to achieve lower mean accuracy across datasets than the other algorithms.

**Table 3:** Value of accuracy and sensitivity for each algorithm.

	**OPSO**	**PSO**	**MTBO**	**DE**	**ACO**	**CGO**	**LSHADE**	**AOA**
**Mean of accuracy**								
D1	0.9237	0.7857	0.7286	0.7000	0.7000	0.7571	0.8143	0.7429
D2	0.9591	0.7286	0.7000	0.6571	0.6000	0.7143	0.7286	0.7286
D3	0.9229	0.8429	0.8000	0.8000	0.8000	0.8000	0.8143	0.8000
D4	0.9357	0.8690	0.8333	0.8095	0.8214	0.8333	0.9286	0.9286
D5	0.9214	0.9000	0.8000	0.8000	0.8000	0.8857	0.7857	0.8000
D6	0.9214	0.9143	0.8286	0.8143	0.7857	0.9000	0.9000	0.8429
**STD of accuracy**								
D1	0.0247	0.0082	0.0165	0.0000	0.0082	0.0660	0.0383	0.0495
D2	0.0308	0.0330	0.1016	0.0660	0.0459	0.0308	0.0473	0.1028
D3	0.0165	0.0308	0.0082	0.0000	0.0082	0.0000	0.0082	0.0082
D4	0.0275	0.0257	0.0137	0.0000	0.0137	0.0069	0.0710	0.0770
D5	0.0742	0.0957	0.0495	0.0000	0.0000	0.0742	0.0710	0.0708
D6	0.0884	0.0555	0.0412	0.0165	0.0000	0.0571	0.0825	0.0247
**Mean of sensitivity**								
D1	0.9483	0.9143	0.7714	0.7143	0.8857	0.8857	0.8857	0.8000
D2	0.9429	0.8571	0.8000	0.8000	0.8000	0.8000	0.8857	0.8286
D3	0.9800	1.0000	0.9429	0.9429	0.9714	0.9429	0.9714	0.9714
D4	0.9833	1.0000	0.9524	0.9048	0.9762	0.9524	0.9143	0.9143
D5	0.9686	0.9429	0.7143	0.7143	0.7143	1.0000	0.8000	0.7714
D6	0.9546	0.9714	0.8571	0.8286	0.9143	0.9714	**1.0000**	0.8857
**STD of sensitivity**								
D1	0.0000	0.0825	0.0330	0.0000	0.1155	0.1320	0.0921	0.0495
D2	0.0165	0.0781	0.0921	0.0451	0.0766	0.0165	0.1090	0.1170
D3	0.0000	0.0330	0.0165	0.0000	0.0000	0.0000	0.0165	0.0165
D4	0.0000	0.0275	0.0275	0.0000	0.0137	0.0000	0.0781	0.0946
D5	0.0000	0.1485	0.0165	0.0000	0.0165	0.1650	0.1090	0.0495
D6	0.0165	0.0660	0.0165	0.0000	0.0825	0.0660	0.1096	0.0433

Bold values indicate the best performance compared to the other values.

**Table 4:** Value of AUC, and F1-score for each algorithm.

	**OPSO**	**PSO**	**MTBO**	**DE**	**ACO**	**CGO**	**LSHADE**	**AOA**
**Mean of sensitivity**								
D1	0.9483	0.9143	0.7714	0.7143	0.8857	0.8857	0.8857	0.8000
D2	0.9429	0.8571	0.8000	0.8000	0.8000	0.8000	0.8857	0.8286
D3	0.9800	1.0000	0.9429	0.9429	0.9714	0.9429	0.9714	0.9714
D4	0.9833	1.0000	0.9524	0.9048	0.9762	0.9524	0.9143	0.9143
D5	0.9686	0.9429	0.7143	0.7143	0.7143	1.0000	0.8000	0.7714
D6	0.9546	0.9714	0.8571	0.8286	0.9143	0.9714	**1.0000**	0.8857
**STD of sensitivity**								
D1	0.0000	0.0825	0.0330	0.0000	0.1155	0.1320	0.0921	0.0495
D2	0.0165	0.0781	0.0921	0.0451	0.0766	0.0165	0.1090	0.1170
D3	0.0000	0.0330	0.0165	0.0000	0.0000	0.0000	0.0165	0.0165
D4	0.0000	0.0275	0.0275	0.0000	0.0137	0.0000	0.0781	0.0946
D5	0.0000	0.1485	0.0165	0.0000	0.0165	0.1650	0.1090	0.0495
D6	0.0165	0.0660	0.0165	0.0000	0.0825	0.0660	0.1096	0.0433
**Mean of AUC**								
D1	0.9523	0.8367	0.7895	0.8401	0.8071	0.7255	0.7238	0.8354
D2	0.9591	0.8133	0.7867	0.7432	0.5224	0.6173	0.5680	0.8133
D3	0.9557	0.9194	0.9000	0.8844	0.8418	0.8847	0.8847	0.8857
D4	0.9630	0.9328	0.9167	0.9036	0.8682	0.9039	0.9872	0.9530
D5	0.9414	0.7929	0.7548	0.7779	0.6714	0.7582	0.7371	0.8180
D6	0.9663	0.9122	0.8711	0.8677	0.7847	0.8531	0.8490	0.8687
**STD OF AUC**								
D1	0.0000	0.0473	0.0077	0.0076	0.0350	0.0076	0.0077	0.0558
D2	0.0082	0.0297	0.0715	0.0824	0.1917	0.0824	0.0715	0.0885
D3	0.0082	0.0281	0.0000	0.0024	0.0377	0.0024	0.0000	0.0317
D4	0.0069	0.0234	0.0000	0.0020	0.0314	0.0020	0.0000	0.0264
D5	0.0000	0.0399	0.0024	0.0124	0.1290	0.0124	0.0024	0.0824
D6	0.0082	0.0393	0.0068	0.0078	0.0761	0.0078	0.0068	0.0446
**Mean of F1-Score**								
D1	0.9151	0.8114	0.7375	0.6925	0.6899	0.7886	0.7682	0.7201
D2	0.9598	0.7639	0.7345	0.7112	0.6509	0.7317	0.7840	0.7025
D3	0.9241	0.8707	0.8321	0.8321	0.8264	0.8290	0.8340	0.8288
D4	0.9367	0.8923	0.8601	0.8323	0.8325	0.8575	0.9450	0.9013
D5	0.9208	0.9045	0.8786	0.8786	0.8786	0.8967	0.8110	0.8505
D6	0.9343	0.9198	0.8317	0.8160	0.7931	0.9046	0.8513	0.8234
**STD of F1-Score**								
D1	0.0000	0.0285	0.0230	0.0000	0.0044	0.1112	0.0778	0.0526
D2	0.0089	0.0406	0.0303	0.0523	0.0000	0.0167	0.1171	0.1664
D3	0.0067	0.0270	0.0085	0.0000	0.0099	0.0000	0.0087	0.0085
D4	0.0056	0.0225	0.0145	0.0000	0.0169	0.0064	0.0072	0.0145
D5	0.0000	0.0567	0.0111	0.0000	0.0000	0.0312	0.0775	0.0376
D6	0.0107	0.0477	0.0344	0.0132	0.0000	0.0492	0.0773	0.0247

Bold values indicate the best performance compared to the other values.

**Table 5:** Fitness value metrics for each algorithm.

	**OPSO**	**PSO**	**MTBO**	**DE**	**ACO**	**CGO**	**LSHADE**	**AOA**
**Mean of fitness**								
D1	**0.1722**	0.3392	0.3297	0.3750	0.3791	0.1851	0.2066	0.2883
D2	0.1637	**0.1412**	0.3053	0.3624	0.3661	0.1677	0.1849	0.2547
D3	0.1123	0.3236	0.2270	0.2728	0.2762	**0.1120**	0.1383	0.1892
D4	0.0938	0.3007	0.1974	0.2466	0.2500	0.0936	**0.0587**	0.0605
D5	0.0530	0.2362	0.1398	0.2132	0.2338	**0.0443**	0.0626	0.0943
D6	0.0542	0.2190	0.2025	0.2642	0.2681	**0.0378**	0.0507	0.1259
**STD of fitness**								
D1	0.0031	0.0481	0.0023	0.0013	**0.0004**	0.0302	0.0428	0.0083
D2	0.0148	0.0149	0.0150	0.0012	**0.0005**	0.0149	0.0298	0.0084
D3	0.0084	0.0205	0.0024	0.0010	**0.0004**	0.0076	0.0279	0.0082
D4	0.0085	0.0172	**0.0026**	0.0068	0.0064	0.0065	0.0430	0.0567
D5	**0.0035**	0.0245	0.0147	0.0078	0.0151	0.0258	0.0350	0.0825
D6	0.0097	0.0164	**0.0027**	0.0077	0.0074	0.0100	0.0153	0.1195
**Minimum of fitness**								
D1	**0.1292**	0.3363	0.3276	0.3738	0.3787	0.1548	0.1676	0.2828
D2	0.1549	**0.1295**	0.2916	0.3612	0.3655	0.1548	0.1677	0.2455
D3	**0.0907**	0.3142	0.2244	0.2718	0.2758	0.1033	0.1163	0.1799
D4	0.0757	0.2921	0.1948	0.2421	0.2460	0.0862	0.0179	**0.0042**
D5	0.0267	0.2333	0.1267	0.2085	0.2249	0.0272	0.0240	**0.0118**
D6	0.0401	0.2091	0.2002	0.2595	0.2636	0.0274	0.0310	**0.0094**
**Worst of fitness**								
D1	0.2195	0.3422	0.3321	0.3762	0.3795	**0.2071**	0.2455	0.2975
D2	0.1809	**0.1576**	0.3182	0.3634	0.3665	0.1806	0.2194	0.2604
D3	0.1296	0.3296	0.2289	0.2736	0.2766	**0.1164**	0.1685	0.1942
D4	0.1084	0.3075	0.1997	0.2542	0.2573	0.0975	0.0775	**0.0646**
D5	0.0729	0.2400	0.1529	0.2221	0.2512	**0.0722**	0.0768	0.1036
D6	0.0688	0.2281	0.2053	0.2729	0.2765	**0.0452**	0.0701	0.1367

Bold values indicate the best performance compared to the other values.

Moreover, the standard deviations of the DE and ACO algorithms are consistent across some datasets. For example, DE achieved a value of 0.0000 on three datasets (i.e., D3, D4, and D5) and the ACO algorithm in D1 and D3, indicating consistent results across experiments. Conversely, the OPSO algorithm exhibits higher variability in performance on D5 (0.0742) and D6 (0.0884), indicating lower stability on these datasets despite its high accuracy. Furthermore, the PSO, CGO, and LSHADE algorithms achieved outstanding performance, with sensitivity reaching an optimal value of 1.00 for PSO in D4, CGO in D5, and LSHADE in D6. As with accuracy, the OPSO and DE algorithms show the highest levels of consistency in sensitivity, with OPSO achieving a value of 0.0000 in D1, D3, and D4, and DE achieving the same value in D3, D4, and D5. Conversely, the ACO, CGO, and LSHADE algorithms show significant variability across some datasets, such as ACO in D1 (0.1155) and CGO in D5 (0.1650), indicating that their sensitivity results are less stable.

**Figure 4:**
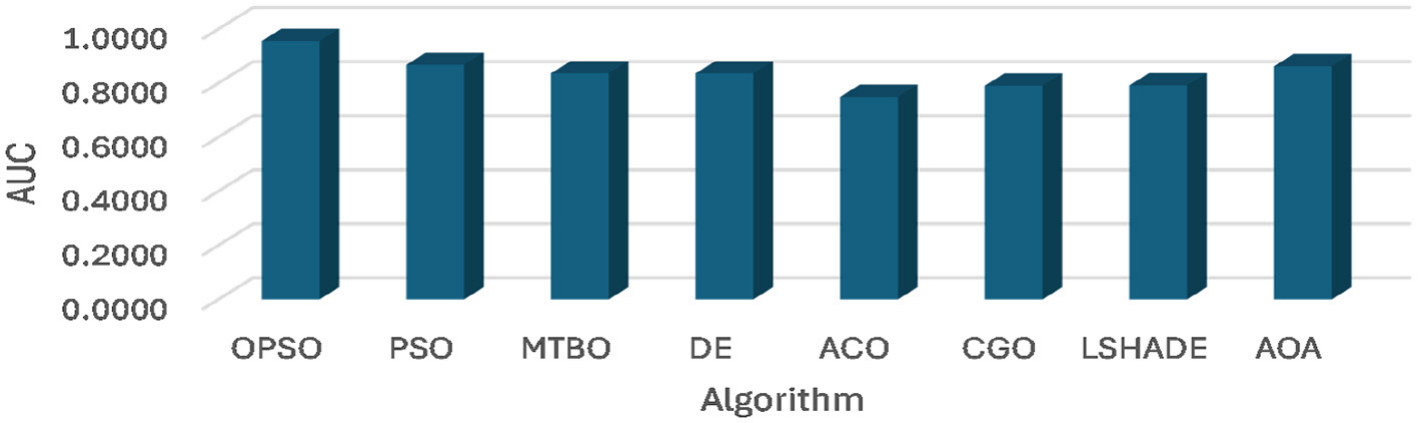
Average of AUC obtained using OPSO and other algorithms.

**Figure 5:**
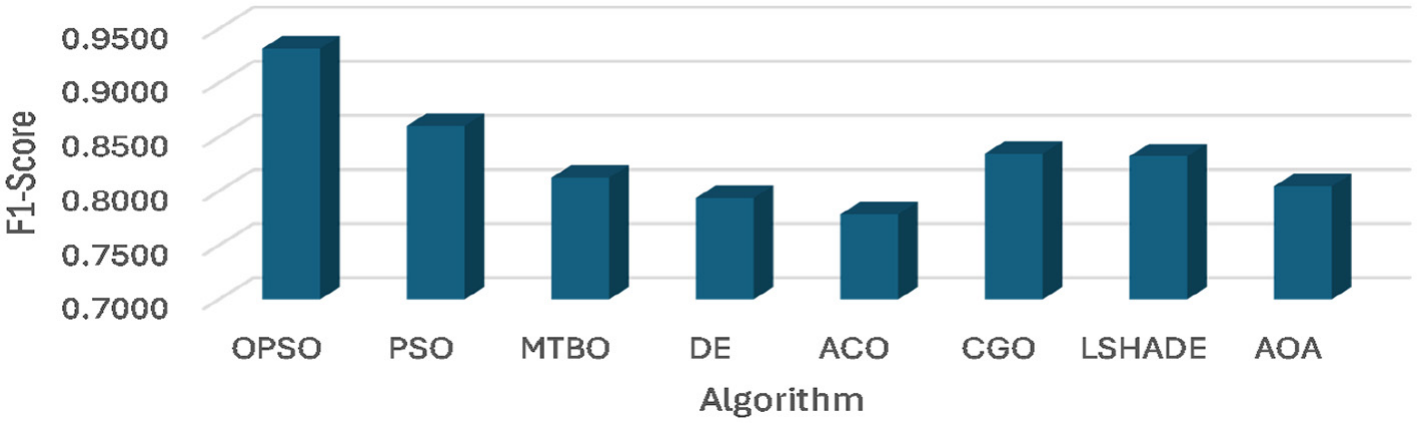
Average of F1 score obtained using OPSO and other algorithms.

Table 4 and Figures 4, 5 show the average of AUC and F1-score for each algorithm. From these results, it is clear that the OPSO algorithm leads, achieving the highest mean AUC across all six datasets (D1 to D6). Its values ranged from 0.9414 to 0.9663, demonstrating its superior discriminative ability. However, the CGO and LSHADE algorithms recorded the lowest mean AUC values across most datasets. For example, in D1, CGO, and LSHADE achieved the lowest values (0.7255 and 0.7238, respectively), indicating weaker discriminative ability than OPSO.

The STD AUC of the algorithms shows that the OPSO algorithm is consistent, recording the lowest STD across three datasets (D1, D5, and D6), with values of 0.0000 in D1 and D5. LSHADE also achieved the lowest STD across D1, D3, and D4, including a value of 0.0000 in D3 and D4. However, PSO and ACO algorithms record the highest STD values in most cases, especially ACO in D2 (0.1917), indicating significant variability and instability in their discrimination performance across experiments.

Based on the average F1 score, the OPSO performs best overall, achieving the highest mean F1 score across three datasets (D1, D2, and D3), indicating an effective balance between precision and recall. Whereas LSHADE demonstrates strong competition, achieving the highest F1-score in D4 and D6. CGO also achieved the highest value in D5 (0.8967). From the STD of F1-Score, it can be noticed that the DE algorithm showed high consistency, achieving a value of 0.0000 across three datasets (D3, D4, and D5), demonstrating the stability of its results in achieving taxonomic balance. ACO also achieved a value of 0.0000 in D3, D5, and D6. However, CGO, LSHADE, and AOA recorded the highest variance values in some cases, with CGO in D1 (0.1112), LSHADE in D2 (0.1171), and AOA in D6 (0.0376).

**Figure 6:**
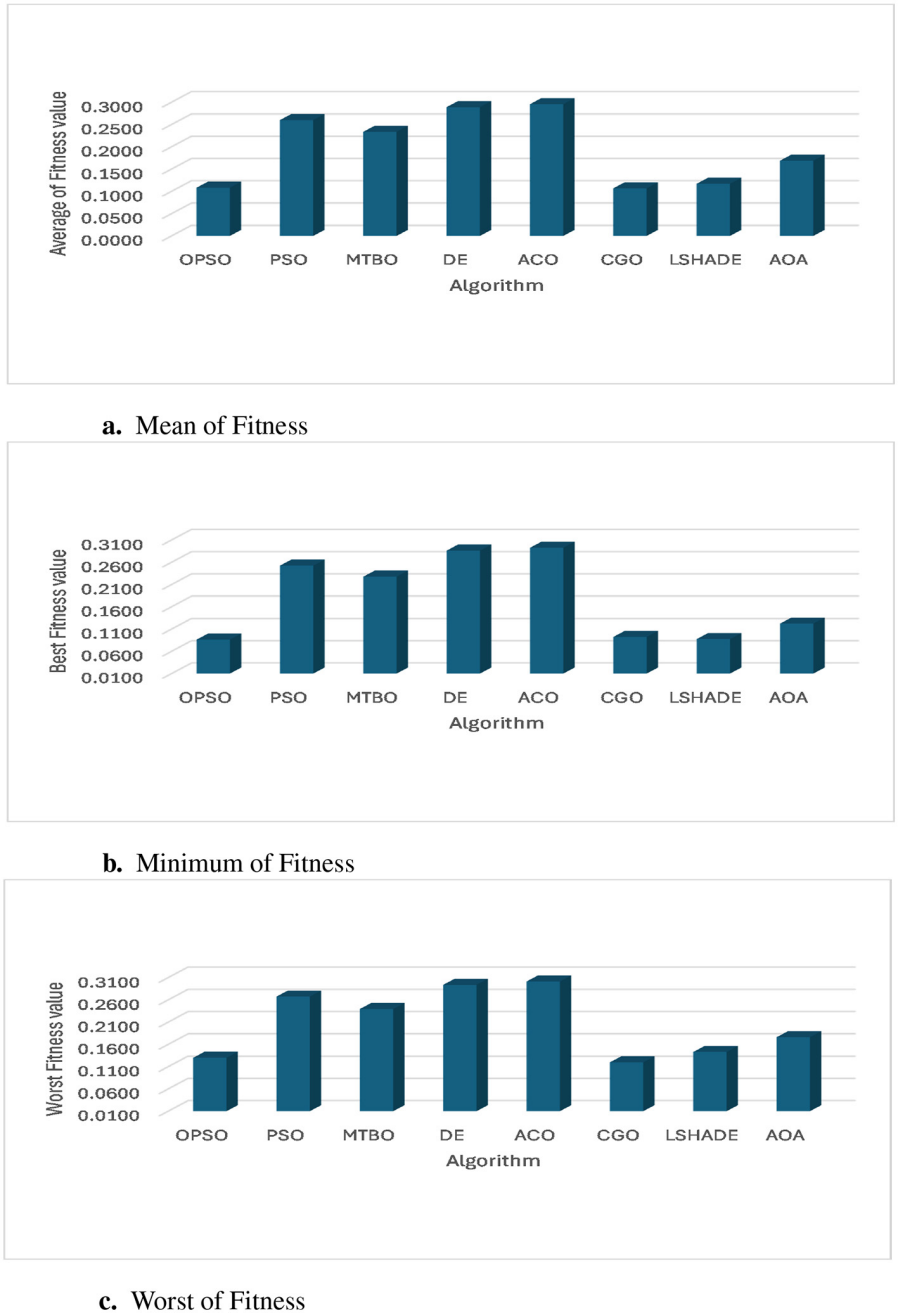
Performance metrics based on fitness value: **(a)** Mean, **(b)** Minimum, and **(c)** Maximum of fitness value.

To further analyze the performance of the developed model, Table 5 and Figure 6 show the metrics of fitness value for each algorithm. From these results, it is clear that the CGO algorithm clearly outperforms the algorithm in achieving the lowest mean fitness across most datasets (D2, D3, D4, D5, and D6), indicating its high effectiveness in minimizing fitness across trials. On dataset D1, PSO achieved the lowest mean (0.1722). Furthermore, the DE and ACO algorithms frequently recorded the highest mean fitness values, meaning their solutions were lower on average than those of other algorithms.

The ACO algorithm shows the highest consistency, recording the lowest STD of fitness value across three datasets (D1, D2, and D3). MTBO also recorded the lowest deviation in D4 and D5. Very low STD (such as 0.0004 for ACO) indicates that the algorithm produces solutions of similar quality each time the experiment is run. In some cases, AOA, PSO, and LSHADE algorithms record the highest STD of fitness values, indicating that the quality of their solutions varies more with the initial conditions of the experiment.

Based on the minimum fitness value, the CGO algorithm outperforms in finding the best solutions (lowest minimum fitness values) in most datasets (D2, D3, D4, D5, D6). Interestingly, the AOA algorithm achieved the lowest absolute value in D4 (0.0042) and D6 (0.0094). According to the worst fitness value, the CGO algorithm continues to outperform, achieving the lowest worst-fit values across four datasets (D2, D3, D4, D5). This reinforces the idea that CGO rarely produces solutions of very poor quality. On D6, LSHADE achieved the lowest value (0.0103).

To further assess the results obtained using the proposed model, we used a nonparametric test named the Friedman test at a confidence interval 95%. Table 6 shows the mean rank obtained using the Friedman test. From these values of mean rank, we can observe that the highest mean rank is achieved using OPSO in terms of accuracy, sensitivity, AUC, and F1-score. The PSO is the second-best algorithm in these metrics. However, the CGO has the lowest mean rank and the worst fitness value, followed by the proposed OPSO algorithm.

**Table 6:** Mean Rank obtained using the Friedman test for each algorithm over the performance metrics.

	**OPSO**	**PSO**	**MTBO**	**DE**	**ACO**	**CGO**	**LSHADE**	**AOA**	***P*-value**
Accuracy	8	6.33	3.16	2.16	2	4.5	5.33	4.5	3.72E-05
Sensitivity	7	6.91	2.5	1.58	4.08	4.75	5.41	3.75	2.35E-04
AUC	7.8	6.25	4.5	4	1.5	3.08	3.08	5.75	1.38E-04
F1-score	7.83	6.5	4.25	2.75	1.66	4.66	5.33	3	1.16E-04
Mean fitness	2.33	6.16	5.16	6.5	7.5	1.83	2.66	3.83	4.36E-05
Min fitness	2.5	6.16	5.16	6.5	7.5	2.66	2.83	2.66	1.07E-04
Worst fitness	2.5	6	5.16	6.5	7.66	1.5	3	3.66	3.05E-05

**Figure 7:**
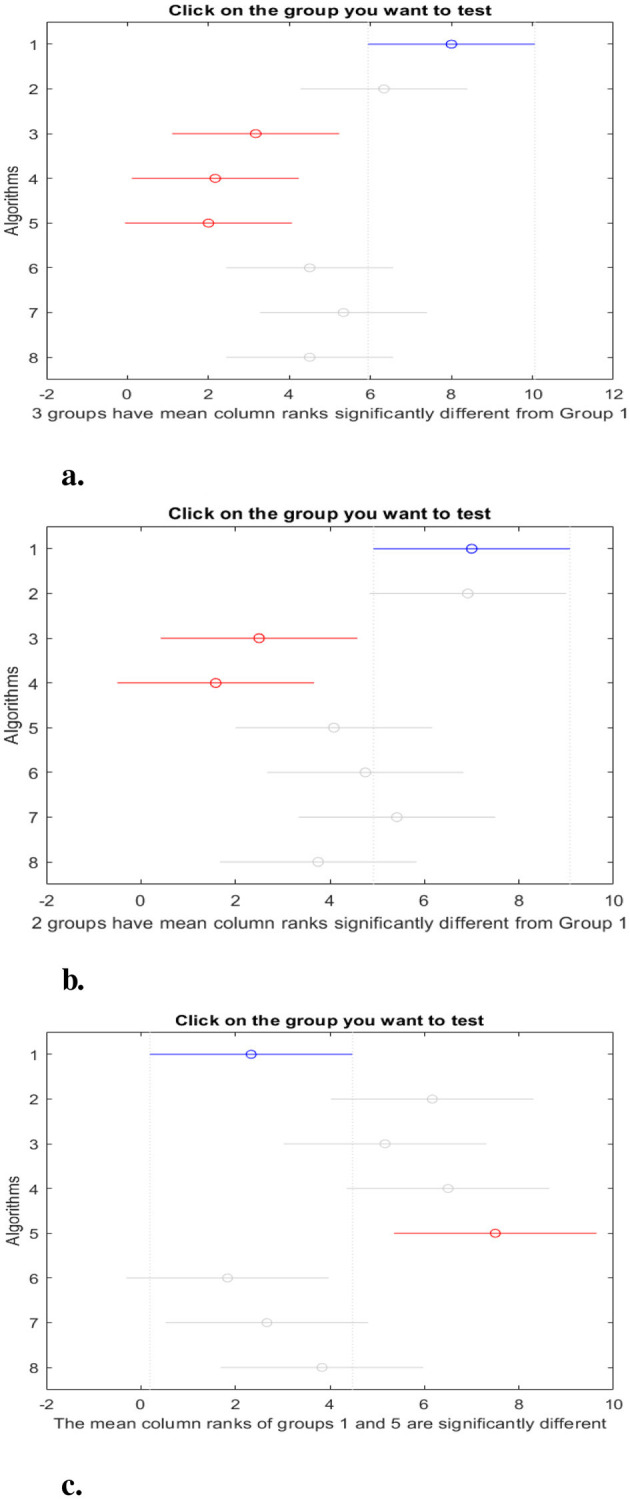
*Post-hoc* test: **(a)** Accuracy, **(b)** Sensitivity, and **(c)** Mean of fitness value. Groups 1 to 8 represent OPSO, PSO, MTBO, DE, ACO, CGO, LSHADE, and AOA, respectively.

**Figure 8:**
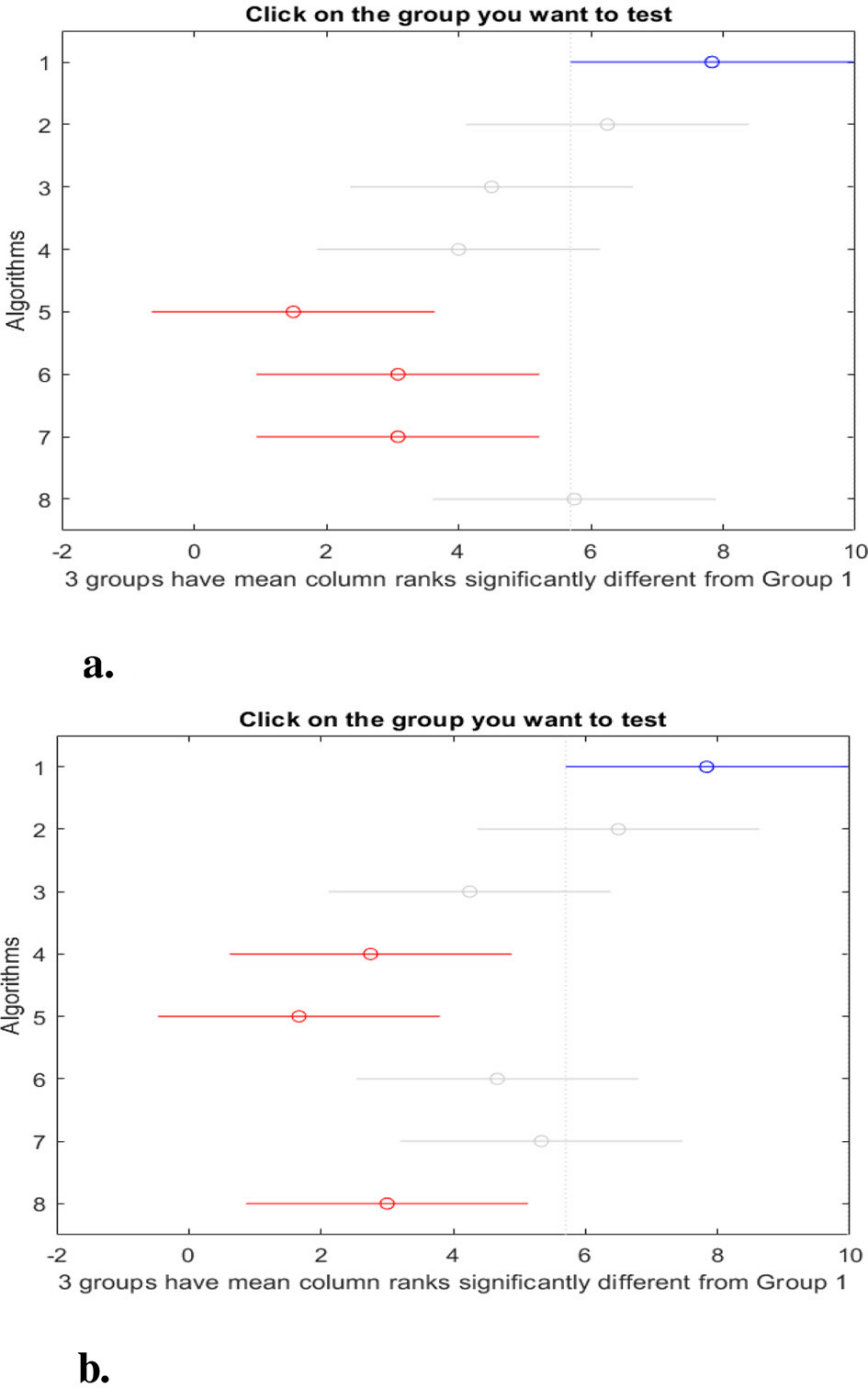
*Post-hoc* test: **(a)** AUC, **(b)** F1 score. Groups 1 to 8 represent OPSO, PSO, MTBO, DE, ACO, CGO, LSHADE, and AOA respectively.

Figures 7, 8 show the *post-hoc* comparison between the proposed model and others using the Nemenyi test. From these figures, we observe a significant difference in accuracy between the proposed model (group 1) and MTBO (group 3), DE (group 4), and ACO (group 5). In terms of sensitivity, there is a significant difference between the proposed model and MTBO (group 3) and DE (group 4). The results of the *post-hoc* test using Fitness value indicate there is a significant difference between the proposed model and ACO (group 5). The results of the AUC indicate a significant difference between OPSO (group 1) and ACO (group 5), CGO (group 6), and LSHADE (group 7). Finally, the *post-hoc* test for F1-score indicates a significant difference between OPSO and DE (group 4), ACO (group 5), and AOA (group 8).

### 6.4 Ablation study

In this section, we presented a comparison between the developed model and other studies that used the same ETDD70 dataset. This comparison is presented in Table 7, and we use the accuracy metric because it is common across studies. From the results given in Table 7, it is clear that (Nguyen et al., 2025) reports accuracy values that vary from 73% to 80.6% for different classifier techniques such as CatBoost, LR, TabPFN, and LLMs. Whereas the classifiers (e.g., SVM, Random Forest, k-NN, and Gradient Boosting) used in Sedmidubsky et al. (2024) nearly have an accuracy of 90%, as well as the accuracy given in Svaricek et al. (2025) using INSIGHT is 86.65%. The average accuracy obtained using CatBoost and XGBoost in Nora (2025) is 80%. Finally, the developed OPSO has the highest accuracy value, which is 94.3%, and this indicates its superiority over all previously reported techniques.

**Table 7:** Comparison between OPSO and other studies using the ETDD70 dataset.

**References**	**Model used**	**Accuracy**
(Nguyen et al. 2025)	CatBoost	73%
	LR	78%
	TabPFN	69%
	Large Language Models (LLMs)	80.6%
(Sedmidubsky et al. 2024)	SVM, Random Forest, k-NN, Gradient Boosting	90%
(Svaricek et al. 2025)	INSIGHT	86.65%
(Nora 2025)	CatBoost and XGBoost	80%
**Our proposed OPSO**		**94.30%**

Finally, this study presented Optical PSO (OPSO), an advanced form of PSO that draws on optical computing principles, used here to select key features for detecting dyslexia from the ETDD70 eye-tracking dataset of 70 Czech children (50 with dyslexia). OPSO delivered better results across six datasets (D1–D6) from different reading tasks, with average accuracy, sensitivity, AUC, and F1-score. These outperformed PSO, ACO, DE, and other methods, as shown by the Friedman test rankings. The findings enable reliable early detection of dyslexia through eye-movement signs such as prolonged fixations and backward jumps. Moreover, there is no one algorithm, including OPSO, that can reach high accuracy among the tested datasets (D1-D6). This aligned with the no-free-lunch theory (NLF), which states that no single algorithm can solve all optimization problems (e.g., feature selection) with the same performance. Furthermore, the proposed model has the following effects on Children with Dyslexia as follows:

- OPSO has sensitivity (up to 98.33%) and AUC (over 0.94), reducing missed cases, which is supported during key brain development years (ages 9–10 in this data), helping to curb lasting issues in reading, school success, and emotional well-being.
- Simple eye-tracking paired with OPSO analysis (94.3% average accuracy) takes over from biased expert judgments, making widespread checks possible in places short on resources, like schools.
- Proposed detection model custom plans that target dyslexia signs (long fixations, regressions), offering tailored reading help and possibly easing the burden of dyslexia, which accounts for 80% of learning problems.
- Dyslexia—a brain-based condition affecting 5%–10% of kids and up to 80% of learning troubles—OPSO marks a real step forward in hands-off detection using ETDD70 eye data. It hits 92%–96% accuracy, 94%–98% sensitivity, and leads in AUC/F1 scores against AI models like SVM/RF (90%), nailing dyslexic eye patterns in reading.

Moreover, there are major benefits for dyslexia care as follows:

- High sensitivity avoids overlooking kids at ages when the brain is most flexible (9–10 years), letting proven treatments build reading skills and dodge emotional setbacks, far better than old guesswork methods.
- Eye-tracking with OPSO brings checks to underfunded schools, shortening waits that widen school gaps for dyslexic kids.

From the previous discussion, it can be observed that the OPSO applies to the management of dyslexia. However, the proposed model still has some limitations, such as time complexity, that need to be reduced. Moreover, the selection of parameters of OPSO.

## 7 Conclusion and future works

In this study, we presented a modified optimization model by developing the Optical Particle Swarm Optimization (OPSO) algorithm. The proposed algorithm relies on incorporating an optical-based updating mechanism. To evaluate the performance of the proposed model, two experiments were conducted. In the first experiment, the main objective is to compare the proposed OPSO and traditional PSO for handling global optimization functions. From this experiment, we demonstrated the superiority of OPSO, which achieved the smallest fitness value and converged faster than PSO. Moreover, the second experiment assessed the applicability of the developed OPSO model for detecting dyslexia. The results of OPSO have been compared with other well-known feature selection techniques. The results illustrated the high ability of the OPSO to reduce the number of features and increase the prediction performance. Finally, the results of the two experiments indicate that OPSO not only enhanced optimization accuracy and strong stability compared to the standard PSO algorithm but also possesses superior discriminative ability in dyslexia detection.

Based on the promising results, the proposed OPSO can be applied in future works in different applications. For example, apply it to enhance Autism detection and other disability diseases. Moreover, the use of the optical mechanism integrated into OPSO to solve other optimization problems outside the medical field could also be explored.

## Data Availability

The original contributions presented in the study are included in the article/supplementary material, further inquiries can be directed to the corresponding author.

## References

[B1] Abualigah L. Diabat A. Mirjalili S. Abd Elaziz M. Gandomi A. H. (2021). The arithmetic optimization algorithm. Comput. Methods Appl. Mech. Eng. 376:113609. doi: 10.1016/j.cma.2020.113609

[B2] Brown T. Mann B. Ryder N. Subbiah M. Kaplan J. D. Dhariwal P. . (2020). “Language models are few-shot learners,” in Advances in Neural Information Processing Systems, 1877–1901.

[B3] Chauhan D. Shivani Suganthan, P. N. (2025). Learning strategies for particle swarm optimizer: a critical review and performance analysis. Swarm Evolut. Comput. 98:102048. doi: 10.1016/j.swevo.2025.102048

[B4] Chen X. Tang K. Yang L. (2024). Comprehensive learning particle swarm optimization based on optimal particle recombination. Int. J. Intell. Control Syst. 29, 21–29. doi: 10.62678/IJICS202403.10105

[B5] Dahou A. Chelloug S. A. Alduailij M. Elaziz M. A. (2023). Improved feature selection based on chaos game optimization for social internet of things with a novel deep learning model. Mathematics 11:1032. doi: 10.3390/math11041032

[B6] El Hmimdi A. E. Kapoula Z. Sainte Fare Garnot V. (2024). Deep learning-based detection of learning disorders on a large scale dataset of eye movement records. BioMedInformatics 4, 519–541. doi: 10.3390/biomedinformatics4010029

[B7] Esteva A. Kuprel B. Novoa R. A. Ko J. Swetter S. M. Blau H. M. . (2017). Dermatologist-level classification of skin cancer with deep neural networks. Nature 542, 115–118. doi: 10.1038/nature21056 28117445 PMC8382232

[B8] Faridmehr I. Nehdi M. L. Davoudkhani I. F. Poolad A. (2023). Mountaineering team-based optimization: a novel human-based metaheuristic algorithm. Mathematics 11:1273. doi: 10.3390/math11051273

[B9] Feldmann J. Youngblood N. Karpov M. Gehring H. Li X. Stappers M. . (2021). Parallel convolutional processing using an integrated photonic tensor core. Nature 589, 52–58. doi: 10.1038/s41586-020-03070-1 33408373

[B10] Gallo-Aristizabal J. D. Escobar-Grisales D. Ríos-Urrego C. D. Vargas-Bonilla J. F. García A. M. Orozco-Arroyave J. R. (2025). Towards Parkinson's disease detection through analysis of everyday handwriting. Diagnostics 15:381. doi: 10.3390/diagnostics15030381 39941311 PMC11817311

[B11] Gomolka Z. Zeslawska E. Czuba B. Kondratenko Y. (2024). Diagnosing dyslexia in early school-aged children using the LSTM network and eye tracking technology. Appl. Sci. 14:8007. doi: 10.3390/app14178004

[B12] Grazioso M. Gallese C. Vanneschi L. Nobile M. S. (2025). “A survey of modern hybrid particle swarm optimization algorithms,” in Applications of Evolutionary Computation: 28th European Conference, EvoApplications 2025, Held as Part of EvoStar 2025, Trieste, Italy, April 23–25, 2025, Proceedings, Part II (Berlin, Heidelberg: Springer-Verlag), 107–128. doi: 10.1007/978-3-031-90065-5_7

[B13] Gulshan V. Peng L. Coram M. Stumpe M. C. Wu D. Narayanaswamy A. . (2016). Development and validation of a deep learning algorithm for detection of diabetic retinopathy in retinal fundus photographs. JAMA 316, 2402–2410. doi: 10.1001/jama.2016.17216 27898976

[B14] Hessels R. S. Niehorster D. C. Kemner C. Hooge I. T. (2017). Noise-robust fixation detection in eye movement data: identification by two-means clustering (i2mc). Behav. Res. Methods 49, 1802–1823. doi: 10.3758/s13428-016-0822-1 27800582 PMC5628191

[B15] Jothi Prabha A. Bhargavi R. (2022). Prediction of dyslexia from eye movements using machine learning. IETE J. Res. 68, 814–823. doi: 10.1080/03772063.2019.1622461

[B16] Kashef S. Nezamabadi-pour H. (2015). An advanced aco algorithm for feature subset selection. Neurocomputing 147, 271–279. doi: 10.1016/j.neucom.2014.06.067

[B17] Kennedy J. Eberhart R. (1995). “Particle swarm optimization,” in Proceedings of ICNN'95-International Conference on Neural Networks (IEEE), 1942–1948. doi: 10.1109/ICNN.1995.488968

[B18] Kitayama K.-I. Notomi M. Naruse M. Inoue K. Kawakami S. Uchida A. (2019). Novel frontier of photonics for data processing–photonic accelerator. Apl Photonics 4:090901. doi: 10.1063/1.5108912

[B19] Kues M. Reimer C. Roztocki P. Cortés L. R. Sciara S. Wetzel B. . (2017). On-chip generation of high-dimensional entangled quantum states and their coherent control. Nature 546, 622–626. doi: 10.1038/nature22986 28658228

[B20] LeCun Y. Bengio Y. Hinton G. (2015). Deep learning. Nature 521, 436–444. doi: 10.1038/nature14539 26017442

[B21] Liang J. J. Qin A. K. Suganthan P. N. Baskar S. (2006). Comprehensive learning particle swarm optimizer for globalLiang et optimization of multimodal functions. IEEE Trans. Evolut. Comput. 10, 281–295. doi: 10.1109/TEVC.2005.857610

[B22] Lin A. Sun W. Yu H. Wu G. Tang H. (2019). Adaptive comprehensive learning particle swarm optimization with cooperative archive. Appl. Soft Comput. 77, 533–546. doi: 10.1016/j.asoc.2019.01.047

[B23] Lu K. Saleh B. E. (1990). Theory and design of the liquid crystal TV as an optical spatial phase modulator. Opt. Eng. 29, 240–246. doi: 10.1117/12.55584

[B24] Nafisah I. Mahmoud N. Ewees A. A. Khattap M. G. Dahou A. Alghamdi S. M. . (2025). Deep learning-based feature selection for detection of autism spectrum disorder. Front. Artif. Intell. 8:1594372. doi: 10.3389/frai.2025.1594372 40636395 PMC12237974

[B25] Nerušil B. Polec J. Škunda J. Kačur J. (2021). Eye tracking based dyslexia detection using a holistic approach. Sci. Rep. 11:15687. doi: 10.1038/s41598-021-95275-1 34344972 PMC8333039

[B26] Nguyen Q.-T. Nguyen H. Tang Q.-H. Truong T. Pham V.-T. Le L. . (2025). “Learning disorder detection using eye tracking: are large language models better than machine learning?” in Proceedings of the 2025 Symposium on Eye Tracking Research and Applications, 1–13. doi: 10.1145/3715669.3726785

[B27] Nora F. (2025). A machine learning-driven eye-tracking approach for dyslexia diagnosis: insights from the ETDD70 dataset. Zenodo. doi: 10.5281/zenodo.14840189

[B28] Prabha A. J. Bhargavi R. (2020). Predictive model for dyslexia from fixations and saccadic eye movement events. Comput. Methods Programs Biomed. 195:105538. doi: 10.1016/j.cmpb.2020.105538 32526535

[B29] Priyasri G. R. Devi M. U. (2025). “Early dyslexia detection and classification using residual dense-assisted multi-attention transformer and eye tracking data,” in Proceedings of the International Conference on Smart Health and Intelligent Technologies (ICSHit-2024) (Atlantis Press), 46–63. doi: 10.2991/978-94-6463-704-5_6

[B30] Probol N. Mieskes M. (2024). Autism detection in speech-a survey. arXiv preprint arXiv:2402.12880.

[B31] Rajpurkar P. Irvin J. Zhu K. Yang B. Mehta H. Duan T. . (2017). Chexnet: Radiologist-level pneumonia detection on chest x-rays with deep learning. arXiv preprint arXiv:1711.05225.

[B32] Sedmidubsky J. Dostalova N. Svaricek R. Culemann W. (2024). “ETDD70: eye-tracking dataset for classification of dyslexia using AI-based methods,” in International Conference on Similarity Search and Applications (Cham: Springer Nature), 34–48.

[B33] Shami T. El-Saleh A. Alswaitti M. Al-Tashi Q. Summakieh M. Mirjalili S. (2022). Particle swarm optimization: a comprehensive survey. IEEE Access 10, 10031–10061. doi: 10.1109/ACCESS.2022.3142859

[B34] Silver D. Hubert T. Schrittwieser J. Antonoglou I. Lai M. Guez A. . (2018). A general reinforcement learning algorithm that masters chess, shogi, and go through self-play. Science 362, 1140–1144. doi: 10.1126/science.aar6404 30523106

[B35] Snowling M. J. (2013). Early identification and interventions for dyslexia: a contemporary view. J. Res. Special Educ. Needs 13, 7–14. doi: 10.1111/j.1471-3802.2012.01262.x 26290655 PMC4538781

[B36] Song X.-F. Zhang Y. Gong D.-W. Sun X.-Y. (2021). Feature selection using bare-bones particle swarm optimization with mutual information. Pattern Recognit. 112:107804. doi: 10.1016/j.patcog.2020.107804

[B37] Sun Y. Guo J. Yan K. Di Y. Pan C. Shi B. . (2023). A deep memory bare-bones particle swarm optimization algorithm for single-objective optimization problems. PLoS ONE 18:e0284170. doi: 10.1371/journal.pone.0284170 37267332 PMC10237448

[B38] Svaricek R. Dostalova N. Sedmidubsky J. Cernek A. (2025). Insight: combining fixation visualisations and residual neural networks for dyslexia classification from eye-tracking data. Dyslexia 31:e1801. doi: 10.1002/dys.1801 39843401 PMC11754147

[B39] Tanabe R. Fukunaga A. S. (2014). “Improving the search performance of shade using linear population size reduction,” in 2014 IEEE Congress Evol Comput (CEC) (IEEE), 1658–1665. doi: 10.1109/CEC.2014.6900380

[B40] Tiwari C. Tiwari C. Verma A. (2025). “A review on how AI is revolutionizing healthcare delivery and patient care,” in Proceedings of International Conference on Artificial Intelligence for Innovations in Healthcare Industries (ICSHIT 2024) (Springer Nature), 11. doi: 10.2991/978-94-6463-704-5_3

[B41] Toki E. I. (2024). Using eye-tracking to assess dyslexia: a systematic review of emerging evidence. Educ. Sci. 14:1256. doi: 10.3390/educsci14111256

[B42] Topol E. J. (2019). High-performance medicine: the convergence of human and artificial intelligence. Nat. Med. 25, 44–56. doi: 10.1038/s41591-018-0300-7 30617339

[B43] Wang P. Xue B. Liang J. Zhang M. (2022). Differential evolution-based feature selection: a niching-based multiobjective approach. IEEE Trans. Evol. Comput. 27, 296–310. doi: 10.1109/TEVC.2022.3168052

[B44] Yin L. Shi Z. Liu M. Chen H. (2025). Effect of hit rate and cognitive style on Bayesian reasoning: evidence from eye movements. Front. Psychol. 16:1485283. doi: 10.3389/fpsyg.2025.1485283 40166404 PMC11955681

[B45] Yu X. Qiao Y. (2021). Enhanced comprehensive learning particle swarm optimization with dimensional independent and adaptive parameters. Comput. Intell. Neurosci. 2021:6628564. doi: 10.1155/2021/6628564 33628213 PMC7880717

[B46] Zhao S.-Z. Liang J. Suganthan P. Tasgetiren M. (2008). “Dynamic multi-swarm particle swarm optimizer with local search for large scale global optimization,” in 2008 IEEE Congress on Evolutionary Computation (IEEE World Congress on Computational Intelligence) (IEEE), 3845–3852. doi: 10.1109/CEC.2008.4631320

[B47] Zhao S.-Z. Suganthan P. N. Pan Q.-K. Tasgetiren M. F. (2011). Dynamic multi-swarm particle swarm optimizer with harmony search. Expert Syst. Appl. 38, 3735–3742. doi: 10.1016/j.eswa.2010.09.032

[B48] Zuo Y. Li B. Zhao Y. Jiang Y. Chen Y.-C. Chen P. . (2019). All-optical neural network with nonlinear activation functions. Optica 6, 1132–1137. doi: 10.1364/OPTICA.6.001132

